# Changes in Phosphorylation of the TREM2‐PLCG2 Signaling Pathway Components Influences Microglia Function

**DOI:** 10.1002/alz70855_103535

**Published:** 2025-12-24

**Authors:** Fusheng Chen, Jennifer K Griffin, Ye Zhou, Kanayo Satoh, Deniz Ghaffari, James Henderson, Beatrice Acheson, Emma Mead, Peter St George‐Hyslop

**Affiliations:** ^1^ University of Toronto, Toronto, ON, Canada; ^2^ University of Oxford, Oxford, Oxfordshire, United Kingdom; ^3^ Columbia University, New York, NY, USA

## Abstract

**Background:**

Missense mutations in the TREM2 gene are associated with increased risk of Alzheimer's disease (AD). Aβ is a known ligand of TREM2 binding directly and activating a signalling pathway involving PLCG2. TREM2 itself signals through its association with DAP12 and recruits SYK through its cytosolic immune‐receptor tyrosine‐based activation motifs. This study will provide insights into relevant TREM2‐PLCG2‐associated cellular processes to help to identify novel interacting proteins and dissect cellular mechanisms involved in AD.

**Method:**

Aβ and anti‐TREM2 activating antibody were used to stimulate TREM2 signalling in mouse microglia followed by western blotting to interrogate the phosphorylation state of various interactors under basal and stimulated conditions. Co‐IP and Immunofluorescence studies were performed to investigate protein interactors in this signalling cascade. Migration and Aβ engulfment assays were used as downstream functional readouts of TREM2 stimulation. Results are representative of at least three independent biological replicate experiments.

**Result:**

We discovered that the endogenous TREM2‐DAP12‐PLCG2 signaling complex interacts with numerous novel high affinity components in microglia. We found that the levels of phospho‐SYK (Tyr525/526) and non‐canonical phospho‐tyrosines in PLCG2 were significantly increased by TREM‐2 activation. Increased Phospho‐PLCG2 levels triggered changes in microglia function reflected in functional assays such as cell migration and cargo engulfment assays.

**Conclusion:**

We discovered that stimulation of TREM2 in microglia led to a novel signalling cascade involving PLCG2 and other proteins which mediated important microglia functions. Some of these proteins are components of the B cell receptor signaling machinery acting via PLCG2 while others are known/new AD‐related risk genes. The elucidation of the TREM2‐PLCG2 pathway and its downstream signalling partners provides important insights into the mechanisms of normal microglia function and in the pathogenesis of AD.

